# ANRIL promotes the regulation of colorectal cancer on lymphatic endothelial cells via VEGF-C and is the key target for Pien Tze Huang to inhibit cancer metastasis

**DOI:** 10.1038/s41417-023-00635-w

**Published:** 2023-06-07

**Authors:** Bin Huang, Yao Lu, Zhuona Ni, Jinhong Liu, Yanbin He, Honglin An, Feimin Ye, Jiayu Shen, Minghe Lin, Yong Chen, Jiumao Lin

**Affiliations:** 1 Academy of Integrative Medicine of Fujian University of Traditional Chinese Medicine, 350122 Fuzhou, Fujian P.R. China; 2 Fujian Key Laboratory of Integrative Medicine on Geriatrics, Fujian University of Traditional Chinese Medicine, 350122 Fuzhou, Fujian P.R. China; 3 Key Laboratory of Integrative Medicine of Fujian Province University, Fujian University of Traditional Chinese Medicine, 350122 Fuzhou, Fujian China

**Keywords:** Drug development, Colorectal cancer

## Abstract

lncRNA ANRIL is an oncogene, however the role of ANRIL in the regulation of colorectal cancer on human lymphatic endothelial cells (HLECs) is remain elusive. Pien Tze Huang (PZH, PTH) a Tradition Chinese Medicine (TCM) as an adjunctive medication could inhibit the cancer metastasis, however the mechanism still uncovering. We used network pharmacology, subcutaneous and orthotopic transplanted colorectal tumors models to determine the effect of PZH on tumor metastasis. Differential expressions of ANRIL in colorectal cancer cells, and stimulating the regulation of cancer cells on HLECs by culturing HLECs with cancer cells’ supernatants. Network pharmacology, transcriptomics, and rescue experiments were carried out to verify key targets of PZH. We found PZH interfered with 32.2% of disease genes and 76.7% of pathways, and inhibited the growth of colorectal tumors, liver metastasis, and the expression of ANRIL. The overexpression of ANRIL promoted the regulation of cancer cells on HLECs, leading to lymphangiogenesis, via upregulated VEGF-C secretion, and alleviated the effect of PZH on inhibiting the regulation of cancer cells on HLECs. Transcriptomic, network pharmacology and rescue experiments show that PI3K/AKT pathway is the most important pathway for PZH to affect tumor metastasis via ANRIL. In conclusion, PZH inhibits the regulation of colorectal cancer on HLECs to alleviate tumor lymphangiogenesis and metastasis by downregulating ANRIL dependent PI3K/AKT/VEGF-C pathway.

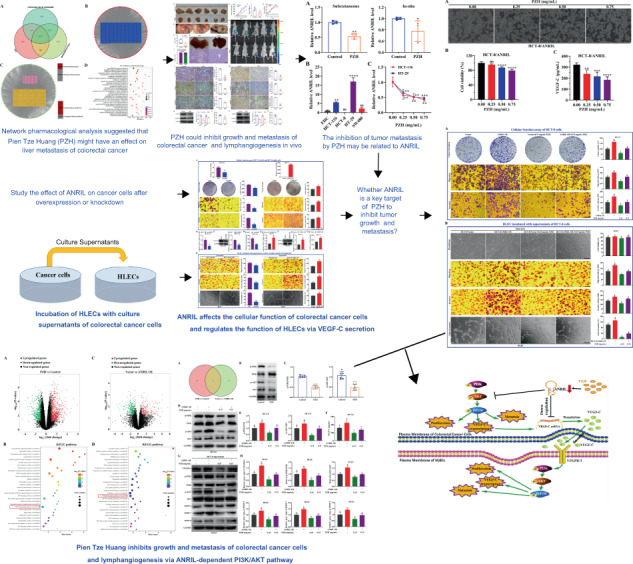

## Introduction

Colorectal cancer (CRC) is one of the most malignant tumors of the digestive tract attributed to modifiable risk factors, including smoking, unhealthy diet, heavy drinking, lack of exercise and being overweight [1]. Since the onset of colorectal cancer is clinically insidious, the early manifestations are mainly changes in bowel movement or indigestion, making early diagnosis difficult [2]. Most patients already lose the best opportunity for surgery when they are diagnosed [3]. In addition, after the patient is treated with radiotherapy and chemotherapy, most patients have recurrence or metastasis, which is the leading cause of death [4]. Colorectal cancer metastasis is the process of cancer cells spreading from the primary tumor through lymph or blood vessels to other tissues and organs [5, 6]. Cancer cells could regulate human lymphatic endothelial cells (HLECs) via cytokine secretion and promote lymphangiogenesis [7], which is a promoter of cancer lymph node metastasis [8]. Studies have shown that the infiltration of intrahepatic lymphatic vessels often predicts poor prognosis and outcome lymph in patients with liver metastases [9].

Vascular endothelial growth factor C (VEGF-C) and vascular endothelial growth factor receptor 3 (VEGFR-3) play an important role in lymphangiogenesis [10, 11]. Lymphatic vessel endothelial hyaluronan receptor-1 (LYVE-1) is one of the specific markers of lymphatic endothelium. Recent studies have found that LYVE-1 can bind to growth factors, such as VEGF-C, and participate in the autocrine and paracrine regulation of tumor-associated HLECs growth and migration [12]. The Phosphatidylinositol-3-kinase (PI3K)/AKT pathway plays a major role in a diverse range of biological processes, including cell proliferation [13]. It is worth noting that, in the cancer cells, PI3K/AKT is the upstream pathway of VEGF-C, and the activation of PI3K/AKT promotes cancer cells’ VEGF-C secretion [14]. However, between cancer cells and HLECs interaction, the PI3K/AKT in HLECs could be activated after the VEGF-C, from cancer cells, binds to its ligand VEGFR-3 on the surface of HLECs [15, 16]. Matrix metalloproteinase (MMP) family is a family of proteases that can degrade extracellular matrix. Following the activation of the PI3K/AKT signaling pathway, the expression of its downstream MMPs is upregulated and the migration of HLECs is promoted by degrading the extracellular matrix, ultimately leading to lymphangiogenesis [17, 18].

Antisense non-coding RNA in the INK4 locus (ANRIL) is a long non-coding RNA (lncRNA) of 3.8 kilobases (kb), which is important for the occurrence of many kinds of cancers [19, 20]. Studies have found that ANRIL plays an important role in tumor metastasis and poor tumor prognosis [20–22]. Therefore, targeting the regulation of ANRIL and its downstream pathway, looking for high-efficiency and low-toxicity inhibitors is full of important scientific value and practical significance for preventing and treating colorectal cancer metastasis and improving the prognosis of patients.

In recent years, it has been found that Traditional Chinese Medicine (TCM) is effective and has little toxicity and side effects in the adjuvant treatment of tumors [10, 23, 24]. Therefore, anti-tumor studies of TCM and its active monomers have become the focus of cancer research. Pien Tze Huang (PZH, PTH), a well-known anti-inflammatory TCM preparation, has been sold in China for 65 years and abroad for 23 years. PZH contains a variety of precious Chinese medicinal materials, such as panax notoginseng, bezoar, snake gallbladder, and musk [25]. The whole prescription of PZH helps clear heat and detoxify, cool the blood and remove blood stasis, reduce swelling, and relieve pain [26]. Modern pharmacological studies have shown that PZH contains a variety of anti-tumor ingredients such as saponins, bilirubin, conjugated cholic acid, muscone, and others, which have anti-tumor, anti-inflammatory, and immune-enhancing effects [27, 28]. We previously found that PZH inhibits colorectal cancer metastasis [11], but whether its efficacy is related to inhibiting tumor lymphangiogenesis through blocking cancer and HLECs interaction have not been elucidated. To find a reliable target of PZH to inhibit colorectal cancer metastasis, and provide a key experimental basis for the clinical use of PZH, we combining the research of ANRIL function with PZH pharmacodynamics, via two tumor-bearing models, colorectal cancer cells model with differential expression of ANRIL, simulating the regulation of cancer cells on HLECs in vitro, and rescue experiments.

## Results

### Network pharmacology showed that PZH may have a significant effect on colorectal cancer liver metastasis

**Fig. 1:**
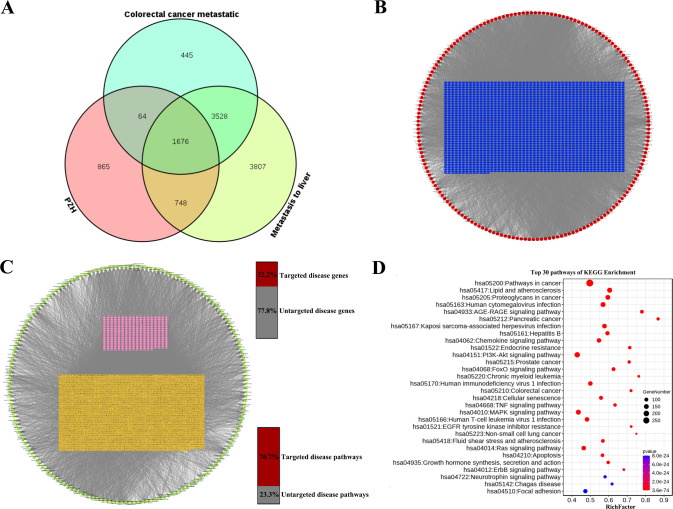
Network pharmacology analysis of the molecular interactions and biological pathways of the PZH in colorectal cancer liver metastasis. **A** Targets attribution among the PZH and colorectal cancer liver metastasis. **B** Disease genes-pathway network. Labels: Blue, disease genes; Red, Pathway. **C** Ingredients-target disease genes-target disease pathway network. Labels: Pink, PZH ingredients; Yellow, target disease genes; Green, target disease pathway. **D** Results of KEGG analysis and pathway analysis in network analysis.

Through the analysis of TCMSP, ETCM, DisGeNET, Drugbank databases, and Kyoto Encyclopedia of Genes and Genomes analyses (KEGG), 3353 drug target genes of PZH, 5204 colorectal cancer liver metastasis genes and 180 colorectal cancer liver metastasis signaling pathway were found (Fig. 1A, B). PZH could interact with 1676 colorectal cancer liver metastasis genes, and 138 disease signaling pathway, it accounted for 32.2% of the disease genes, 76.7% of the disease signaling pathway (Fig. 1C). KEGG results showed that potential target genes were enriched and associated with AGE-RAGE, Chemokine, PI3K-AKT, FoXO, TNF, MAPK, Ras, ErbB, and Neurotrophin signaling pathway, according to the degree of enrichment, range by degree of enrichment (Fig. 1D).

### PZH inhibited the growth and metastasis of transplanted colorectal cancer tumors

**Fig. 2:**
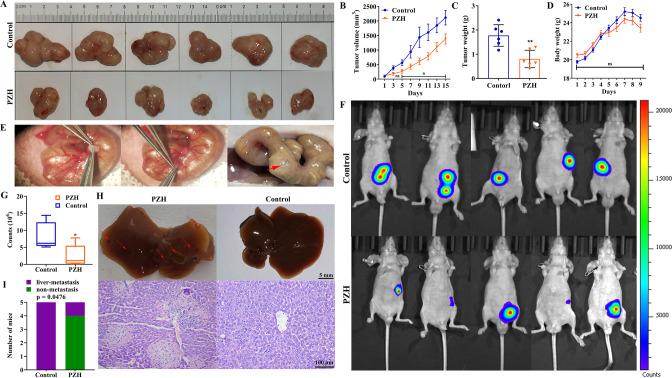
PZH inhibited the growth of colorectal tumors and inhibited cancer liver metastasis. **A** The image of the tumor. **B** Tumor volume with PZH treatment. Data represent the mean ± standard error of the mean (SEM) of experiments conducted on 6 mice. ns, no significance; *P < 0.05. **C** Tumor weight with PZH treatment. Data represent the mean ± SEM of experiments conducted on six mice. *P < 0.05. **D** The curve demonstrating the changes in mouse body weight. Data represent the mean ± SEM of experiments conducted on six mice. ns, no significance. **E**–**G** The tumor fluorescence signal intensity in the orthotopic tumor model with PZH treatment. Data represent the mean ± SEM of experiments conducted on 5 mice. *^,^ **P < 0.05. **H**, **I** PZH inhibited the liver metastasis of orthotopic transplantation tumors, in comparison with the control group.

We successfully constructed an animal model of HCT-116 cell subcutaneous xenograft tumor and HCT-116/luc cell orthotopic xenograft tumor. The tumor in the PZH group was significantly reduced compared with the control group. The volume of tumor was statistically different from the 5th day (*P* < 0.05) (Fig. 2A–C). The average weight of the tumor in the control group (1.77 ± 0.45 g) was statistically different in the PZH group (0.8 ± 0.36 g) (*P* < 0.01) compared to the control group. Whereas, there was no significant difference in the body weight of nude mice between the control group and the PZH group (*P* > 0.05) (Fig. 2D).

The HCT-116/luc orthotopic xenograft tumor model showed liver metastasis. The results of IVIS Spectrum Imaging System showed that, compared with the PZH group, the fluorescence signal intensity and volume of the tumor in the orthotopic xenograft tumor model group (control group) were stronger (*P* < 0.05), which reflected that PZH significantly reduced tumor formation and growth (Fig. 2E–G). To further verify if PZH inhibits the metastasis of colorectal cancer, liver metastasis and hepatocyte morphology of nude mice were observed anatomically and using HE staining. The liver of the control group mice had obvious metastases, while the liver of the PZH group mice had a smooth texture and ruddy color (Fig. 2H). The liver metastasis rate of the control group was 100%, whereas that of the PZH group was 20% (Fig. 2I), which was significantly lower than that of the control group (*P* < 0.05). HE staining results showed that the liver cells in the control group had obvious necrosis and inflammatory cell infiltration, with disordered structure of hepatic lobules and irregular arrangement of hepatocytes, while in the PZH group, there was no edema or necrosis, accompanied with normal structure of hepatic lobules, obvious demarcation, neat arrangement of hepatocyte elements, and no infiltration of inflammatory cells (Fig. 2H).

### PZH inhibited the expression of LYVE-1, VEGF-C, and VEGFR-3

**Fig. 3:**
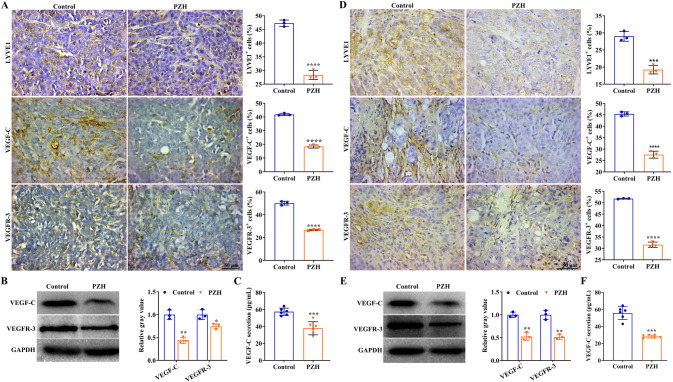
PZH inhibited LYVE-1, VEGF-C, and VEGFR-3 expression in vivo. **A**, **D** Expression of LYVE-1, VEGF-C, and VEGFR-3 in subcutaneous xenograft tumor tissues (**A**) and orthotopic xenograft tumor tissues (**D**), Data represent the mean ± SEM of experiments conducted on 3 mice. ***^,^ ****P < 0.01 in comparison with the control group. **B**, **E** Expression of VEGF-C and VEGFR-3 in xenograft tumor tissues (**B**) and orthotopic xenograft tumor tissues (**E**). Data represent the mean ± SEM of experiments conducted on 3 mice. *P < 0.05; **P < 0.01 in comparison with the control group. **C**, **F** VEGF-C content in the serum of mice with subcutaneous xenograft tumor (**C**) and orthotopic xenograft tumor (**F**). Data represent the mean ± SEM of experiments conducted on 6 mice. ***P < 0.01 in comparison with the control group.

The effect of PZH on the expression of VEGF-C, VEGFR-3, and LYVE-1 was detected using IHC in two kinds of animal models, which showed that PZH significantly reduced the expression of LYVE-1, VEGF-C, and VEGFR-3 in subcutaneous xenograft tumor tissues and orthotopic xenograft tumor tissues (*P* < 0.01) (Fig. 3A, D). In the subcutaneous xenograft tumor model, the positive rates (percentage expression) of LYVE-1, VEGF-C and VEGFR-3 in the control group were 47.28 ± 1.17%, 41.84 ± 0.83%, and 50.18 ± 1.71%, respectively, and those in the PZH group were 28.37 ± 1.67%, 18.39 ± 1.11%, and 26.71 ± 0.62%, respectively. In the orthotopic xenograft tumor model, the positive rates of LYVE-1, VEGF-C, and VEGFR-3 in the control group were 29.00 ± 1.42%, 45.41 ± 0.93%, and 51.80 ± 0.19%, respectively, and those in the PZH group were 19.25 ± 1.25%, 27.55 ± 1.52%, and 31.61 ± 1.15%, respectively. Furthermore, western blotting was used to detect the effect of PZH on the expression of VEGF-C and VEGFR-3. PZH significantly inhibited the expression of VEGF-C and VEGFR-3 in tumor tissues (*P* < 0.01, *P* < 0.05) (Fig. 3B, E). We also detected the VEGF-C content in the serum of nude mice using ELISA. PZH significantly reduced the serum VEGF-C content of nude mice with subcutaneous xenografts or orthotopic xenografts (*P* < 0.01) (Fig. 3C, F). In the subcutaneous xenograft tumor model, the average VEGF-C serum levels of the two groups were 56.49 ± 4.57 pg/mL for the control group and 37.87 ± 7.69 pg/mL for the PZH group. In orthotopic xenograft tumor models, the average VEGF-C serum levels of the two groups were 54.0 ± 8.31 pg/mL for the control group and 27.87 ± 1.73 pg/mL for the PZH group.

### PZH interfered with the expression of ANRIL in vivo and in vitro

**Fig. 4:**
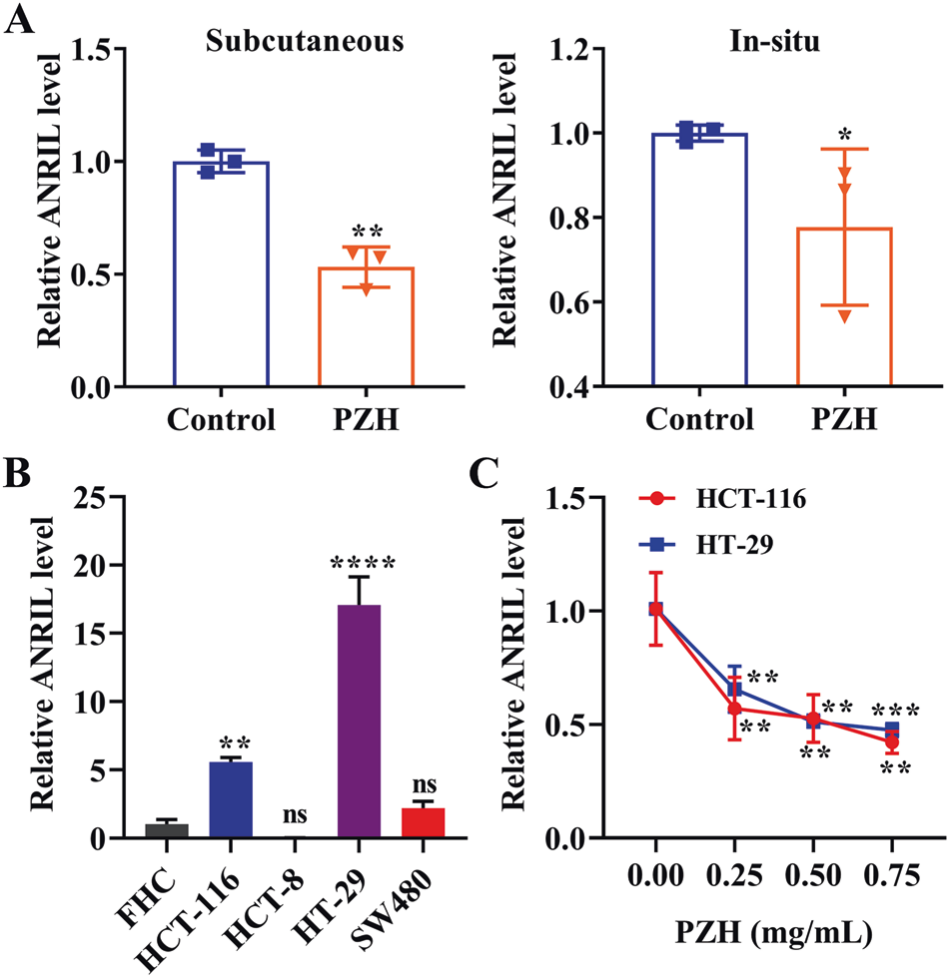
PZH inhibited the expression of ANRIL in vivo and in vitro. **A** The expression of ANRIL in the subcutaneous xenograft tumor model and orthotopic xenograft tumor model with PZH treatment. Data represent the mean ± SEM of experiments conducted on 3 mice. *P < 0.05; **P < 0.01. **B** ANRIL expression in different colorectal cancer cell lines. Data represent the mean ± SEM of experiments conducted in triplicate. ns, no significance; **^,^ ****P < 0.01 in comparison with FHC. **C** The expression of ANRIL in HCT-116 and HT-29 cells with different concentrations of PZH treatment. Data represent the mean ± SEM of experiments conducted in triplicate. **^,^ ***P < 0.01 in comparison with 0.00 mg/mL PZH.

PZH significantly decreased the expression of ANRIL in subcutaneous xenograft (0.47 folds) and orthotopic xenograft tumor tissues (0.22 folds) (*P* < 0.01) (Fig. 4A). Furthermore, PZH could downregulate ANRIL in HCT-116 and HT-29 cells via concentration dependence (Fig. 4B, C). These results reflected that the mechanism of lymphangiogenesis inhibition in colorectal cancer by PZH may be related to the inhibition of the ANRIL expression.

### ANRIL overexpression or knockdown affected the regulation of colorectal cancer cells on HLECs via VEGF-C secretion

**Fig. 5:**
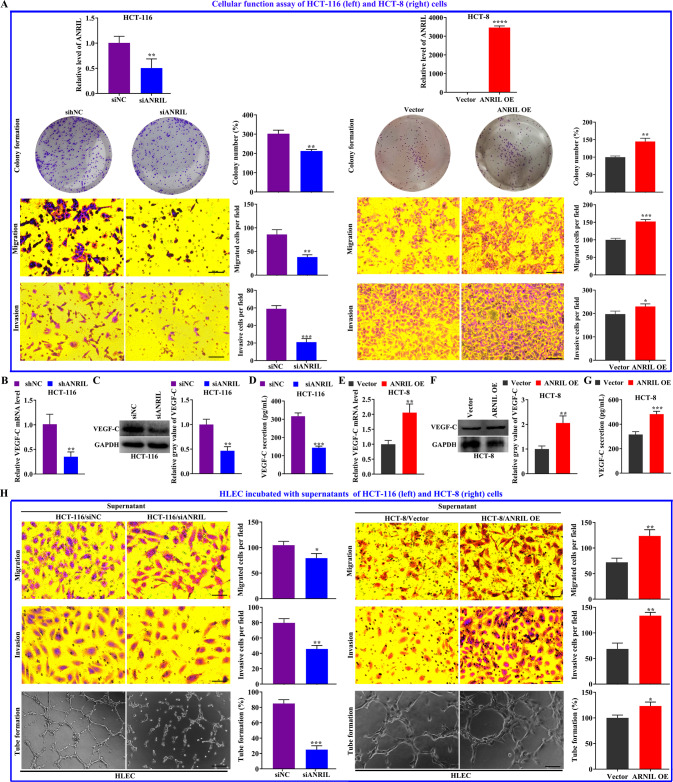
ANRIL affected the cellular function of colorectal cancer cells and the regulation of cancer cells on HLECs via VEGF-C secretion. **A** The ANRIL expression, colony formation, migration and invasion abilities of cancer cells with ANRIL knockdown (HCT-116/siANRIL, compare with HCT-116/NC) or overexpression (HCT-8/ANRIL, compared with HCT-8/Vector). **B**–**D** The VEGF-C expression at mRNA level (**B**), at protein level in cancer cells (**C**) and in the supernatant (**D**) of HCT-116 cells with ANRIL knockdown (HCT-116/siANRIL, compared with HCT-116/NC). **E**–**G** The VEGF-C expression at mRNA level (**E**), at protein level in cancer cells (**F**) and in the supernatant (**G**) of HCT-8 cells with ANRIL overexpression (HCT-8/ANRIL, compare with HCT-8/Vector). **H** The migration, invasion and tube formation abilities of cancer cells with ANRIL knockdown (HCT-116/siANRIL, compare with HCT-116/NC) or overexpression (HCT-8/ANRIL, compare with HCT-8/Vector). Data represent the mean ± SD of experiments conducted in triplicate experiments, *P < 0.05; **^,^ ***^,^ ****P < 0.01.

Simulating the regulation of cancer cells on HLECs in vitro by incubating the HLECs with colorectal cancer cells’ supernatant. We successfully constructed the HCT-8/ANRIL and HCT-116/siANRIL cell models, and found that overexpression of ANRIL promoted the proliferation, migration, invasion (Fig. 5A), and the secretion of VEGF-C (*P* < 0.01) of HCT-8 cells, while downregulation on ANRIL had the opposite effect on HCT-116 (Fig. 5B–G). The supernatant of HCT-8/ANRIL and HCT-116/siANRIL cells was collected and used to culture HLECs for 24 h. The migration, invasion, and tube formation of HLECs treated with the HCT-8/ANRIL cells’ supernatant was higher than that of the HCT-8/Vector cells (*P* < 0.05) (Fig. 5H). In contrast, the migration, invasion, and tube formation of HLECs treated with the HCT-116/siANRIL cells’ supernatant was lower than that of the HCT-116/NC group (*P* < 0.05) (Fig. 5H).

### Overexpression of ANRIL decreased the effect of PZH on inhibiting cancer cells and the regulation of cancer cells on HLECs

**Fig. 6:**
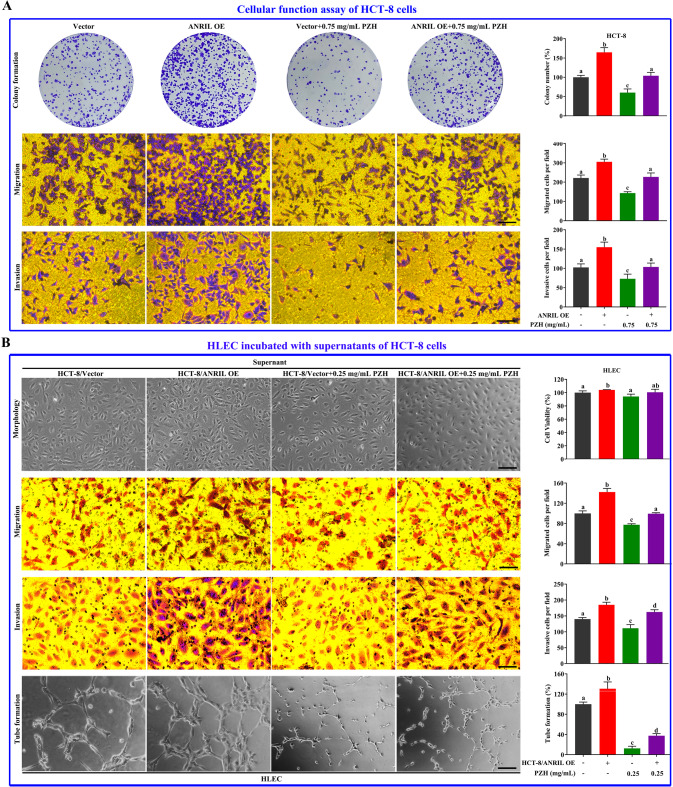
Overexpression of ANRIL decreased the effect of PZH on cancer cells, and the regulation of cancer cells on HLECs. **A** The colony formation, migration, invasion abilities of HCT-8/ANRIL cells with or without 0.75 mg/mL PZH treatment, compared with HCT-8/Vector cells with or without 0.75 mg/mL PZH treatment. **B** The cell viability, migration, invasion and tube formation abilities of HLECs incubated with supernatants of HCT-8/ANRIL cells with or without 0.25 mg/mL PZH treatment, compared with HCT-8/Vector cells with or without 0.25 mg/mL PZH treatment. Data represent the mean ± SD of experiments conducted in triplicate experiments. One-way ANOVA followed by Tukey’s test was used to evaluate the statistical significance, different letters represent significant differences between different groups (*P* < 0.05).

We found PZH inhibited HCT-8/ANRIL via concentration dependence. 0.25 mg/mL PZH have no influence on inhibiting HCT-8/ANRIL cell viability (*P* > 0.05), and 0.5 or 0.75 mg/mL PZH could inhibit cancer cells viability (*P* < 0.05) (Fig. S1A, B). While, it is worth noting that, all concentrations (0.25, 0.5, 0.75 mg/mL) of PZH could inhibit VEGF-C secretion (vs. control, *P* < 0.05), and there was no difference in the effect of the three different concentrations (*P* > 0.05) (Fig. S1C). To further verify whether ANRIL is a key target of PZH, we carried out rescue experiments in vitro, and chose 0.75 mg/mL PZH to incubate HCT-8/ANRIL. We found overexpression of ANRIL promoted cell HCT-8 cell colony formation (*P* < 0.01), migration (*P* < 0.01) and invasion (*P* < 0.05), and alleviated the inhibitory effect of PZH on cancer cells (*P* < 0.05) (Fig. 6A).

Our focus is on the regulation of cancer cells on HLECs mediated by VEGF-C. In order to avoid the release of other cytokines due to PZH-induced cancer cell death to interfere the role of VEGF-C, we chose 0.25 mg/mL PZH to treat the cancer cells. After HCT-8/Vector or HCT-8/ANRIL cells were treated with 0.25 mg/mL PZH for 24 h, the supernatant was collected and used to culture HLECs for 24 h. There were four supernatant treatment groups as follows: HCT-8/Vector supernatant group, HCT-8/Vector+PZH supernatant group, HCT-8/ANRIL supernatant group, and HCT-8/ANRIL + PZH supernatant group. The morphological observation and MTT results showed that the density and viability of HLECs with the HCT-8/ANRIL cell supernatant treatment were increased compared with the HCT-8/Vector group (*P* < 0.05), but the density and viability of HLECs cultured by HCT-8/ANRIL + PZH or HCT-8/Vector + PZH supernatant did not decrease significantly (*P*å 0.05) (Fig. 6B).

Migration, invasion and tube formation of HLECs treated with the HCT-8/ANRIL cell supernatant was higher than that of the HCT-8/Vector group (*P* < 0.05). Compared with the HCT-8/ANRIL and HCT-8/Vector groups, the migration and invasion of HLECs treated with HCT-8/ANRIL + PZH or HCT-8/Vector+PZH supernatant decreased significantly (*P* < 0.05). The migration, invasion and tube formation of HLECs with HCT-8/ANRIL + PZH supernatant treatment was higher than that in the HCT-8/Vector + PZH supernatant group (*P* < 0.05). For HCT-8/Vector + PZH and HCT-8/ANRIL + PZH supernatant groups: 77.48 ± 1.987 and 99.67 ± 1.515 cells migration per field, respectively; 111.3 ± 11.37 and 162.3 ± 7.095 cells invasion per field, respectively; 12.50 ± 4.17 and 37.50 ± 4.17% of tube formation rate, respectively (Fig. 6B).

### Transcriptomic show that PI3K/AKT signaling pathway is a key pathway for ANRIL and PZH to affect colorectal cancer metastasis

**Fig. 7:**
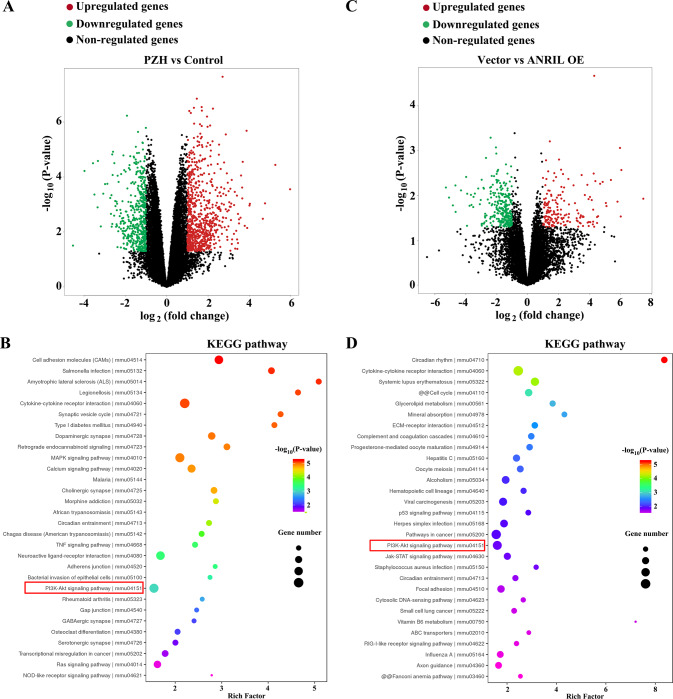
Transcriptomics analysis changes of transcriptional level in PZH-treated tumor-bearing mice with orthotopic tumor transplantation, and HCT-8 cells with ANRIL overexpression. **A** Volcano plots was used to compare gene expression profiles in Control (Model) vs. PZH (Model + PZH) (|fold change| ≥ 1.5, *P* < 0.05). **B** Pathway enrichment analysis was performed to identify the related Reactome pathways in Control (Model) vs. PZH (Model + PZH). **C** Volcano plots was used to compare gene expression profiles in Vector (HCT-8/Vector) vs. ANRIL OE (HCT-8/ANRIL) (|fold change| ≥ 1.5, *P* < 0.05). **D** The pathway enrichment analysis was performed to identify the related Reactome pathways in Vector (HCT-8/Vector) vs. ANRIL OE (HCT-8/ANRIL).

Transcriptomics technology was used to detect mRNA expression in tumor tissues of the orthotopic tumor models, as well as in HCT-8/ANRIL and HCT-8/Vector cells. Kyoto Encyclopedia of Genes and Genomes (KEGG) enrichment analysis showed that PZH inhibited colorectal cancer metastasis related to p53, PI3K-AKT, Jak-STAT, and RIG-I-like receptor signaling pathways (Fig. 7A, B). ANRIL increased the migration, invasion, and tube formation of HCT-8 cells related to MAPK, TNF, PI3K/AKT, and NOD-like receptor signaling pathways (Fig. 7C, D).

### The PI3K/AKT pathway is the most important pathway for PZH to affect tumor metastasis via ANRIL

**Fig. 8:**
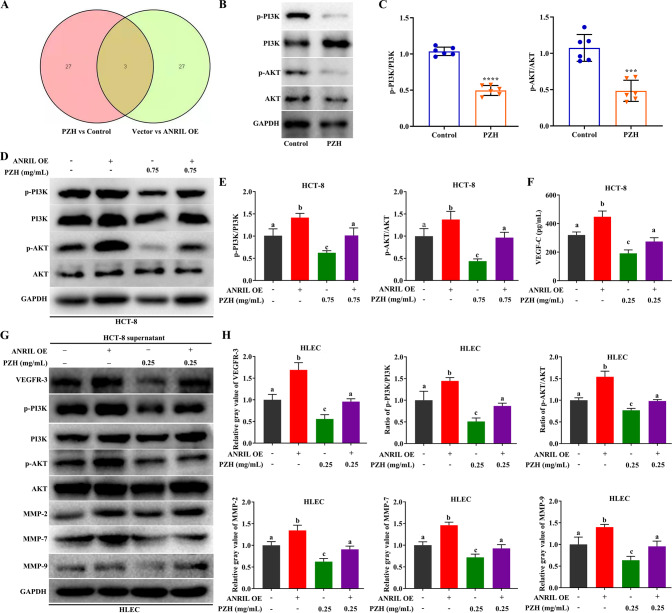
The PI3K/AKT pathway is the most important pathway for PZH to affect tumor metastasis via ANRIL. **A** Analyzing the intersection of two transcriptomic results. **B**, **C** p-PI3K/PI3K and p-AKT/AKT ratios in orthotopic xenograft tumors with or without PZH treatment. Data represent the mean ± SEM of experiments conducted in triplicate. ***^,^ ****P < 0.01. **D**, **E** p-PI3K/PI3K and p-AKT/AKT ratios in HCT-8/ANRIL cells with or without 0.75 mg/mL PZH treatment, compared with HCT-8/Vector cells with or without 0.75 mg/mL PZH treatment. **F** The concentration of VEGF-C in supernatant of HCT-8/ANRIL with or without 0.25 mg/mL PZH treatment, compared with HCT-8/Vector cells with or without 0.25 mg/mL PZH treatment. **G**, **H** p-PI3K/PI3Kratios, p-AKT/AKT ratios, MMP-2, MMP-7 and MMP-9 expression of HLECs incubated with supernatants of HCT-8/ANRIL cells with or without 0.25 mg/mL PZH treatment, compared with HCT-8/Vector cells with or without 0.25 mg/mL PZH treatment. Data represent the mean ± SD of experiments conducted in triplicate. One-way ANOVA followed by Tukey’s test was used to evaluate the statistical significance, different letters represent significant differences between different groups (*P* < 0.05).

We performed an intersection analysis of two transcriptomic results, and found a total of three intersecting biological pathways as follows: Cytokine-cytokine receptor interaction, circadian entrainment, and PI3K/AKT signaling pathway (Fig. 8A). These findings suggested that, ANRIL was the key target of PZH to exert its pharmacodynamic effects on inhibiting cancer cells and HLECs interaction, and PI3K/AKT was the downstream pathway.

We further examined the activation of PI3K/AKT signaling pathway with ANRIL overexpression and PZH treatment in vitro and in vivo. Compared with the control group, p-PI3K/PI3K and p-AKT/AKT ratios in the orthotopic tumor tissues, were decreased with 0.75 mg/mL PZH treatment (Fig. 8B, C). The p-PI3K/PI3K and p-AKT/AKT ratios in the HCT-8/ANRIL group were significantly increased (compared with the HCT-8/Vector group, *P* < 0.05), while these ratios were significantly decreased when cancer cells were treated with PZH (*P* < 0.05). Meanwhile, the inhibitory effect of PZH on PI3K/AKT activation were attenuated after ANRIL overexpression (HCT-8/Vector + PZH group vs HCT-8/ANRIL + PZH group, *P* < 0.05) (Fig. 8D, E).

We examined the VEGF-C content in cancer cell supernatant by using ELISA, and found that the VEGF-C content in the supernatant of HCT-8/ANRIL cells were significantly higher than that in the culture supernatant of HCT-8/Vector cells (*P* < 0.05), while it was significantly decreased when HCT-8/Vector cells were treated with PZH (*P* < 0.05), and the inhibitory effect of PZH on VEGF-C secretion was attenuated with ANRIL overexpression (HCT-8/Vector+PZH group vs HCT-8/ANRIL + PZH group, *P* < 0.05) (Fig. 8F).

Then, we detected the protein expression of VEGFR-3, p-PI3K/PI3K, p-AKT/AKT, MMP-2, MMP-7, and MMP-9. Compared with the HCT-8/Vector supernatant group, p-PI3K/PI3K and p-AKT/AKT ratios and the expression of VEGFR-3, MMP-2, MMP-7, and MMP-9 in HLECs with HCT-8/ANRIL cell supernatant treatment were significantly increased (*P* < 0.05), while these were significantly decreased when HCT-8/Vector cells were treated with PZH (*P* < 0.05). Furthermore, the inhibitory effect of PZH on PI3K/AKT activation, VEGFR-3, MMP-2, MMP-7 and MMP-9 expression were attenuated with ANRIL overexpression (HCT-8/Vector+PZH vs HCT-8/ANRIL + PZH supernatant groups, *P* < 0.05) (Fig. 8G, H).

## Discussion

Colorectal cancer is the third most commonly diagnosed cancer worldwide [29], and the second in China [1, 30]. Although surgery combined with radiotherapy and chemotherapy has significantly improved the five-year survival rate of patients with colorectal cancer, many patients still relapse or the tumor metastasizes after treatment. The reason is that most of the current anti-tumor drugs are single treatment methods and are more likely to have side effects, leading to drug resistance, metastasis, and death [1]. Therefore, the development of multi-component, multi-target, and low-toxicity drugs has become the key to improving the quality of life and five-year survival rate of patients with colorectal cancer.

PZH is one of the most famous TCM preparations and consists of several precious medicinal materials, such as Notoginseng Radix et Rhizoma, Bovis Calculus, Snake Gall, and Moschus. PZH clears away heat, detoxifies, cools the blood and removes blood stasis, reduces swelling, and relieves pain [31]. At present, PZH has not only shown therapeutic effects on hepatitis and colitis in clinical trials but has also been shown to inhibit the growth of colorectal cancer cells and angiogenesis by regulating multiple signaling pathways [32]. However, the study on the efficacy of PZH in inhibiting colorectal cancer metastasis is not comprehensive, and the key mechanism is still unclear, the further research is urgently needed. In this study, the network pharmacology showed that PZH could interfere with 32.2% of the colorectal cancer liver metastasis genes and 77.2% of the disease signaling pathways, which means PZH may have a significant effect on colorectal cancer liver metastasis. A nude mouse subcutaneous xenograft model and orthotopic xenograft model were constructed and treated with PZH. We found that PZH inhibited tumor growth in the subcutaneous xenograft tumor models, and inhibited the fluorescence intensity (reflecting the tumor volume and size) of orthotopic transplantation tumor and liver metastasis.

Cancer cells regulate HLECs by secreting cytokines such as VEGF-C, which is the key to tumor lymph node metastasis [7, 10]. Tumor-related lymphangiogenesis is a promoter of tumor lymph node metastasis, which helps to promote the recruitment of cancer cells to lymph nodes [33]. Tumor lymph node metastasis is the earliest and most common mode of tumor metastasis, and is a sign of advanced cancer and often indicates poor prognosis [34]. The liver is the most common metastatic site for patients with advanced colorectal cancer [35]. In addition, studies have shown that lymphatic metastasis are closely related to liver metastasis of colorectal cancer [36]. VEGF-C is the first identified lymphangiogenic growth factor, which was secretion by cancer cells and specifically bind with its homologous receptor VEGFR-3, and promote the proliferation and migration of HLECs and the formation of the lymphatic lumen, participating in tumor metastasis [7, 10, 37]. Therefore, downregulating the expression of key proteins, such as VEGF-C may significantly inhibit tumor lymph node metastasis [37]. LYVE-1 is a specific marker of HLECs, which can reflect the density of tumor-related lymphatics. LYVE-1 transports hyaluronic acid from tissues to the lymphatic system and regulates the migration of tumor cells in and out of lymphatic vessels, making it closely related to tumor lymphangiogenesis [14, 38, 39]. Since PZH inhibits tumor metastasis, we examined the expression of VEGF-C, VEGFR-3, and LYVE-1, and found that PZH downregulated these genes expression, indicating that PZH could inhibit tumor lymphangiogenesis in colorectal cancer.

Compared with other non-coding RNAs, lncRNAs play an important regulatory role in cell growth and can serve as proto-oncogenes or tumor suppressor genes to participate in the regulation of tumor cell metastasis [40, 41]. The lncRNA ANRIL is upregulated in various malignant tumors, and high expression of ANRIL often leads to poor prognosis of patients [42]. In this study, we constructed cell models differentially expressing ANRIL, and found that overexpression of ANRIL promoted cancer cell viability, colony formation, migration, invasion. Because tumor metastasis is not only associated with the metastatic capacity of the cancer cells itself, but also with the lymphangiogenesis [7], and colorectal cancer cells can regulate HLECs lymphangiogenesis [43], we stimulated the regulation of cancer cells on HLECs by culturing HLECs with cancer cells supernatant. By culturing HLECs with HCT-8/ANRIL cell supernatant, we found that the tube formation of HLECs was significantly increased, compared with HCT-8/NC. However, the tube formation of HLECs cultured with HCT-116/siANRIL supernatant was significantly decreased, compared with HCT-116/NC. Cause VEFG-C, as a lymphangiogenic growth factor, could specifically bind with its homologous receptor VEGFR-3 to promote the formation of the lymphatic lumen of HLECs [37]. In combination, we found an increased concentration of VEGF-C in the supernatant of cancer cells with higher ANRIL expression, these results suggests that the overexpression of ANRIL can promote the VEGF-C secretion and the VEGF-C in supernatant stimulate the lymphangiogenesis of HLECs. Moreover, in vivo experiments, we found that PZH significantly inhibited the expression of ANRIL in both the subcutaneous xenograft model and the orthotopic xenograft model, and PZH showed a concentration dependence ability of inhibiting ANRIL in vitro. We conducted rescue experiments in both HCT-8 cells and HLECs, and found that PZH inhibited the migration, invasion and VEGF-C secretion of HCT-8 cells, and inhibited cancer cells supernatant-mediated HLECs lymphangiogenesis, while these inhibitory effects of PZH was attenuated by ANRIL overexpression. These results suggest that ANRIL is one of the key targets of PZH on inhibiting cancer and HLECs interaction and tumor metastasis.

Through experiments, we have demonstrated that PZH can inhibit HLEC lymphangiogenesis by inhibiting ANRIL and reducing the secretion of VEGF-C of tumor cells. However, the downstream pathway after PZH inhibiting ANRIL is unknown. To clarify the downstream pathway. As a TCM preparations, the pharmacodynamic effect of PZH is the result of multi-target pathway comprehensive action. So, we identified the most important pathway via transcriptomic and KEGG pathway enrichment analyses. We first examined the mRNA transcriptome changes of orthotopic transplantation tumors with or without PZH treatment. Further, we examined the mRNA transcriptome changes of colorectal cancer cells with or without ANRIL high expression. Therefore, the intersection pathway of the two transcriptome analysis results maybe the important pathway influenced by both PZH and ANRIL, and it may be the downstream pathway after PZH inhibiting ANRIL. In the end, we found a total of three intersecting pathways as follows: Cytokine-cytokine receptor interaction, circadian entrainment, and PI3K/AKT signaling pathway. These findings suggested that PI3K/AKT maybe an important downstream pathway after PZH inhibiting ANRIL. Current studies on PI3K/AKT signaling pathway involves in cell proliferation and tumor metastasis, and its expression is dysregulated in various tumors [44, 45]. Therefore, we selected PI3K/AKT for validation study.

The activation of PI3K/AKT pathway promotes cancer cells VEGF-C secretion [14]. The VEGF-C, from cancer cells, could bind to VEGFR-3 on the surface of HLECs, then activates the PI3K/AKT in HLECs [15, 16, 46]. MMPs are downstream cytokines of PI3K/AKT signaling pathway, which degrade the extracellular matrix, allowing cancer cells to invade blood or lymph vessels from the damaged basement membrane, ultimately leading to tumor metastasis [17, 18]. MMP-2 and MMP-9 both belong to gelatinases, also known as gelatin hydrolases [47]. The catalytic domain contains a collagen-binding domain, which can degrade the basement membrane and type IV collagen, thereby promoting tumor metastasis. MMP-7 is a matrix lytic enzyme, also known as matrix lysin, which is mainly secreted by epithelial cells as an enzyme prototype [48]. After activation, substrate specificity increases, leading to matrix degradation and promoting cell migration, invasion, and metastasis [49, 50]. We found that overexpression of ANRIL can promote the activation of PI3K/AKT pathway, the secretion of VEGF-C of colorectal cancer cells. The supernatants of cancer cells with ANRIL overexpression could stimulate the lymphangiogenesis of HLECs. Combining with the previous studies, the reason maybe the overexpression of ANRIL promote the VEGF-C secretion of cancer cells by activating PI3K/AKT pathway, and the VEGF-C in supernatant stimulate the VEGFR3 expression, PI3K/AKT pathway activation, MMPs expression and lymphangiogenesis of HLECs.

To test whether PI3K/AKT signaling pathway is the important downstream pathway after PZH inhibits ANRIL expression, we used in vitro rescue experiments of ANRIL-overexpressing HCT-8 cell incubated with PZH. PZH could significantly inhibit the activation of PI3K/AKT signaling pathway, VEFG-C secretion of colorectal cancer cells, and the cancer cells’ supernatant with PZH treatment could inhibit the VEGFR3 expression, PI3K/AKT pathway activation, MMPs expression and lymphangiogenesis of HLECs. While, these inhibitory effects of PZH were attenuated with ANRIL overexpression. These evidences could reflect that the efficacy of PZH inhibits the regulation of colorectal cancer on HLECs to alleviate tumor lymphangiogenesis and metastasis by downregulating ANRIL dependent PI3K/AKT/VEGF-C pathway.

## Conclusions

**Fig. 9:**
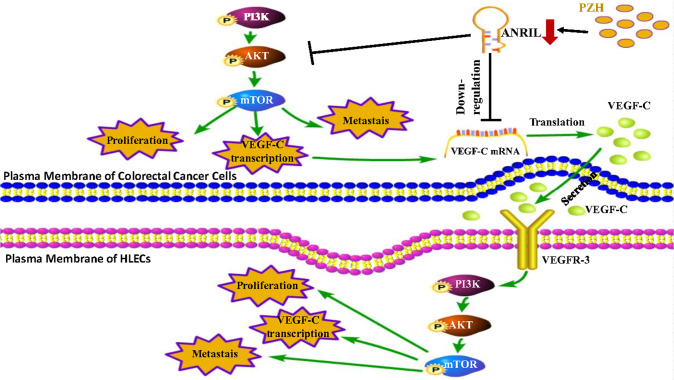
Schematic representation of the mechanism of how PZH inhibits tumor metastasis. Pien Tze Huang inhibits colorectal tumor lymphangiogenesis by regulating the ANRIL dependent PI3K/AKT signaling pathway.

Taken together, we confirmed that PZH inhibits the regulation of colorectal cancer to HLECs on alleviate tumor lymphangiogenesis and metastasis by downregulating ANRIL dependent PI3K/AKT/VEGF-C pathway (Fig. 9). This research provided novel insights regarding the activity of ANRIL promotes colorectal cancer metastasis and contributed to the clinical application of PZH as a potential TCM preparations that targeted ANRIL for colorectal cancer and cancer metastasis.

## Materials and methods

### Network pharmacology studies

#### Related targets of disease and ingredients of the PZH

All protein targets of the PZH ingredients were obtained from the TCMSP (http://tcmspw.com/tcmsp.php), ETCM (http://www.tcmip.cn/ETCM/index.php/Home). Information on the colorectal cancer liver metastasis (the intersection of “colorectal cancer metastatic” and “Metastasis to liver”) was retrieved from the DrugBank (https://www.drugbank.ca), and DisGeNET (https://www.disgenet.org/) databases, and the targets were standardized using the UniProtKB database. Potential targets of the PZH ingredients were obtained by screening for the common targets of the drug and the disease.

#### Protein-protein interaction (PPI) data

Common targets were used to construct the PPI network on the STRING 11.0 platform (https://string-db.org/). The organism was set as “Homo sapiens,” and the minimum required interaction score was 0.4. The topological properties of the PPI network were analyzed using plugins in Cytoscape 3.7.2.

#### Gene pathway analysis

After screening the drug-disease crossover targets, their signal pathways and biological functions were analyzed using Kyoto Encyclopedia of Genes and Genomes (KEGG) (p < 0.05). The enrichment analysis of pathways was conducted using the David database (https://david.ncifcrf.gov/).

#### Network construction

A compound-target-pathway network was constructed by connecting the compounds of the PZH, common crossover targets, and enriched pathways. The network was generated using the interaction network visualization software Cytoscape 3.7.2, which analyzed the molecular interactions and biological pathways of the PZH in colorectal cancer liver metastasis.

### Cell lines and cell culture

Human colorectal cancer cell lines (HCT-116, HCT-8, HT-29, and SW480) were purchased from the Cell Bank of the Chinese Academy of Sciences (Shanghai, China). Human normal intestinal epithelial cells (FHC) and human lymphatic endothelial cells (HLECs) were purchased from the JENNIO Biological Technology Co., Ltd. (Guangzhou, Guangdong, China). These cells have been identified by STR profiling and are free of mycoplasma contamination.

Human colorectal cancer cell lines and FHC cells were cultured in RPMI-1640 medium (C11875500BT, Life Technologies, Carlsbad, CA, USA) supplemented with 10% fetal bovine serum (FBS, 10099141, Life Technologies) and 1% penicillin-streptomycin (SV30010, Life Technologies) at 37 °C in a 5% CO_2_ humidified incubator.

HLECs were cultured in endothelial cell medium (ECM) (SC1001, ScienCell Inc, USA) supplemented with FBS (25 mL, 27849, ScienCell Inc, USA), penicillin-streptomycin (5 mL, 27670, ScienCell Inc, USA), and ECGS (5 mL, 27888, ScienCell Inc, USA) at 37 °C in a 5% CO_2_ humidified incubator.

### PZH preparation

PZH was provided by Zhangzhou Pien Tze Huang Pharmaceutical Co., Ltd. (1808087). PZH was prepared into a 25 mg/mL solution with saline (for in vivo experiments) or phosphate-buffered saline (PBS, for in vitro experiments). After 30 min of ultrasonic-assisted dissolution, the preparation was autoclaved and then sub-packed at −20° C for storage. The original concentration was diluted to the required concentration for the subsequent experiments.

### Cell transfection

We selected HCT-116 cells with a high tumorigenic rate for the construction of luciferase-labeled colorectal cancer cell lines (HCT-116/luc cells) via lentiviral infection and puromycin screening. HCT-116 cells were inoculated onto a six-well plate at a density of 0.4×10^5^ cells/mL, and lentivirus infection was performed when the confluence reached 30%. The multiplicity of infection (MOI) was calculated as 10. The infection mixture was as follows: RPMI 1640 medium, 960 μL; HitransG P infection enhancement solution (25×), 40 μL; lentivirus (1×10^8^, μL), 5 μL. The medium was refreshed after 8 h. Infection efficiency was assessed using fluorescence microscopy 72 h after infection. After two rounds of puromycin screening, 1/10 puromycin concentration was selected to maintain the cultured cells. The lentiviral luciferase kit was purchased from ShanghaiGenechem Co., Ltd.

The expression of ANRIL in HCT-116, HCT-8, HT-29, and SW480 cells was detected using quantitative PCR (qPCR), and FHC was used as the control. The expression of LncRNA ANRIL in HCT-8 cells was significantly lower than that in FHC cells, and HCT-116 and HT-29 cells was significantly higher than that in FHC cells. We chose HCT-116 as the tumor-bearing mouse model cell line, as they had a much higher tumor formation rate than HT-29 cells, and HCT-8 and HCT-116 cells were selected for ANRIL (NR_003529) overexpression or knockdown models.

We constructed HCT-8/ANRIL cells stably overexpressing ANRIL using lentiviral infection (ShanghaiGenechem). MOI was calculated as 10. The infection mixture was as follows: RPMI 1640 medium, 960 μL; HitransG P infection enhancement solution (25×), 40 μL; LV-ANRIL-OE (4×10^8 ^μL), 5 μL; Lentivirus Negative Control (1×10^9^, μL), 2 μL. The transfected cell lines were screened for stable expressions of si-ANRIL by incubating them in a puromycin-containing culture medium for 10 days.

We constructed HCT-116/siANRIL by HCT-116 cells in logarithmic growth phase were inoculated into 6-well plates (2 mL/ well) at the rate of 1.5×10^5^ cells /mL. After 24 h of culture, LncRNA-ANRIL was knocked down with Lipofectamine RNAi MAX transfection reagent. The transfection system was as follows: the final volume of 6-well plates was 1 mL, and the concentrations of Si-NC and Si-ANRIL were 50 nM and 2.5 μL/ well. 6 h after transfection, it was changed to 1640 complete medium to continue culture, and 36 h after transfection, follow-up experiments were done. Sequences of Si-NC and Si-ANRIL were purchased from Shanghai Ji Ma pharmaceutical co., ltd. The sequence of Si-ANRIL was as follows: sense (5′-3′) GCAUAUGUCUUUCUGGUAUT T, antisense (5′-3′) AUACCAGAAAGACAUAUGCTT.

### Animal culture

Male BALB/c nude mice aged 4–5 weeks and weighing 18–20 g (Shanghai SLAC Laboratory Animal Co., Ltd.) were raised in the SPF Laboratory of Experimental Animal Center of Fujian University of Traditional Chinese Medicine. The mice were maintained at a temperature of 25 ± 2 °C and the humidity was controlled at 55%. The mice were kept under 12 h of light every day, eating and drinking normally.

### Subcutaneous xenograft tumor model

HCT-116 cells were prepared into a 2×10^7^ cells/mL single cell suspension in PBS and then mixed with equal volumes of Matrigel, which was inoculated subcutaneously in the right armpit of each mouse (12 nude mice, 0.1 mL/mouse). When the tumor volume reached 100 mm^3^, the mice were randomly divided into control and PZH groups. The control group was given an equal dose of normal saline, while the PZH group was given PZH 250 mg/kg/day. The BALB/c nude mice were administered with normal saline or PZH once daily for 2 weeks. The body weight of mice and the long (a) and short (b) diameters of tumors were measured every other day. The tumor volume was calculated using the following formula: V = π/6×ab^2^. The mice were anesthetized with 2% sodium pentobarbital and blood was collected from the orbit. The blood was centrifuged at 3000 rpm and serum was collected. Finally, the mice were sacrificed with cervical dislocation and tumors were dissected and weighed. The data were not recorded blindly. The tumors were divided into four parts, which were fixed in 4% paraformaldehyde for 48 h and used for transcriptomic analysis, immunohistochemistry (IHC), qPCR, or western blotting.

### Orthotopic xenograft tumor model

HCT-116/luc cells (5×10^6^/200 μL) were inoculated into the bilateral subcutaneous armpits of BALB/c nude mice to construct a subcutaneous xenograft tumor model. After the tumor grew, 50-inoculating needle with an average size of 1 cm^3^ was used to implant the tumor into other nude mice. The tumor tissues of three generations were chosen for orthotopic transplantation into the cecum surface. After the mice were anesthetized with 2% sodium pentobarbital, a small incision was made in the skin above the cecum. Under a dissecting microscope, a 1 mm^3^ xenograft tumor was implanted into the subserosa of the cecum via microsurgery and sutured with 9-0 non-resorbable sutures. Then the cecum was placed back into the abdominal cavity and the peritoneum was sutured with 4-0 absorbable surgical suture. After the mice had recovered, the grouping and dosage were arranged the same as in the subcutaneous xenograft tumor model. Mice were gavaged with normal saline or PZH once a day for 3 weeks, which were randomly assigned and the body weight was measured every other day. IVIS Spectrum Imaging System was used to observe tumor growth and metastasis. After 3 weeks, the mice were anesthetized with 2% sodium pentobarbital and blood was collected from the orbit. The blood was centrifuged at 3000 rpm and serum was collected. Finally, the mice were sacrificed with cervical dislocation and the tumor tissues and liver tissues were peeled off. The number of liver metastases was recorded and photographed with a digital camera. Liver tissues were used for hematoxylin and eosin (HE) staining. Then, the tumors were divided into two groups, which were used for transcriptomic analysis, IHC, and qPCR. The data were not recorded blindly.

### IHC analysis

Three mice in each experimental group were randomly selected. After fixing the tumor tissues in 4% polymethylene formaldehyde for 24 h, they were cut into 4 μm sections. After the waxing was removed, the slides were treated accordingly using KIT-9710 (MXB Biotechnologies, Fuzhou, China). The slides were incubated with the primary antibodies against LYVE-1 (1:200, ab33682, Abcam), VEGF-C (1:100, CST2445, CST, Boston, MA, USA), or VEGFR-3 (1:100, ab27278, Abcam, Shanghai, China). After antibody incubation, slices were stained with DAB (DAB-0031, MXB Biotechnologies) and hematoxylin (G1140, Solaibao, Beijing, China). Finally, three fields were randomly selected under the optical microscope (400×), in which the brown-yellow particles were observed, then analyzed with Motic 6.0 image analysis system.

### Western blotting

Protein was extracted from tumor tissues and cells, and denatured and quantified. Protein samples were then separated via sodium dodecyl sulfate-polyacrylamide gel electrophoresis (SDS-PAGE) electrophoresis and transferred to polyvinylidene fluoride (PVDF) membranes (Roche, Basel, Switzerland). After blocking the membranes with 5% skimmed milk for 2 h, the membranes were incubated with primary antibodies against VEGF-C (1:1000, CST2445, CST, Boston, MA, USA), VEGFR-3 (1:1000, ab27278, Abcam, USA), MMP-2 (1:1000, CST87809S, CST), MMP-7 (1:1000, 10374-2-AP, Proteintech), MMP-9 (1:1000, CST13667S, CST), PI3K (1:1000, 60225-1-1g, Proteintech), p-PI3K (1:1000, ab182651, Abcam), AKT (1:1000, 60203-2-1g, Proteintech), p-AKT (1:1000, 66444-1-1g, Proteintech), or GAPDH (1:5,000, 60004-1-lg, Proteintech) overnight at 4˚ C and subsequently with the appropriate HRP-conjugated secondary antibody followed by enhanced chemiluminescence detection.

### Enzyme-linked immunosorbent assay (ELISA)

HCT-8/ANRIL and HCT-116/siANRIL cells were treated with PZH, and then cell supernatant and mouse serum were collected to detect VEGF-C content. ELISA kits MM-0104M2 and MM-0113H1 (Meimian, Jiangsu, China) were used for the serum and cell supernatant, respectively, following the manufacturer’s instructions.

### Real-time PCR

Total RNA was isolated from tumor tissues or cells with Trizol reagent (R404-01, Vazyme, Nanjing, Jiangsu, China), and then 1000 ng of RNA was converted into cDNA using PrimeScript™ RT Master Mix according to the manufacturer’s instructions (RR047A, TaKaRa, Kyoto, Japan). qPCR was performed using the ABI 7500 Fast PCR system with a SYBR Select Master Mix (A25742, Thermo). The PCR primers used in this study were as follows: ANRIL, forward 5’-CCGCTCC CCTATTCCCCTTA-3’ and reverse 5’-CCTGATTGGCGGATAGAGCA-3’; GAPDH, forward 5’-ATGGGGAAGGTGAAGGTCG-3’ and reverse 5’-GGGGTCATTGATGGCAACAATA-3’.

### HE staining

The liver tissues of the orthotopic xenograft tumor model were fixed with 4% paraformaldehyde for 24 h. After embedding in paraffin, the sections were stained with HE. Histopathological changes were observed under a light microscope.

### Preparation of tumor culture supernatant

HCT-8/ANRIL cells were seeded onto a six-well plate (2 mL/well) at a density of 1.2×10^5^ cells/mL, and after 12 h of culture, they were divided into HCT-8/Vector, HCT-8/ANRIL, HCT-8/Vector + PZH (0.25 mg/mL), and HCT-8/ANRIL + PZH (0.25 mg/mL) groups. HCT-8/Vector and HCT-8/ANRIL groups were grown in complete medium (2 mL/well) alone, whereas HCT-8/Vector + PZH (0.25 mg/mL) and HCT-8/ANRIL + PZH (0.25 mg/mL) groups were grown in the presence of PZH (2 mL/well). After 24 h of treatment, the supernatant was collected. If it was not used immediately, it was frozen in a refrigerator at −80 °C. After thawing, it was centrifuged at 3000 rpm for 15 min to remove cell debris and impurities and used for HLEC culture. The culture supernatant of HCT-116/NC and HCT-116/siANRIL were selected using the same method.

### Observation of morphological changes

HCT-8/ANRIL cells and HLECs cultured in tumor culture supernatant were seeded onto a six-well plate at a density of 1.2×10^5^ and 1.0×10^5^ cells/mL, respectively. After culturing for 24 h, the morphology of the cells was observed using a phase-contrast microscope (Leica Microsystems Co., Ltd., Germany) at 200× magnification.

### Clone formation assay

Cancer cells were inoculated into a 12-well plate at 0.5×10^3^ cells/well (fresh medium every 2 days, for approximately 7 days). When the cells grew to the point where cell clusters could be observed by the naked eye, the cells were washed three times with PBS (1 mL/well), fixed with 4% paraformaldehyde for 20 min (1 mL/well), and stained with 0.1% crystal violet (C8470, Solaibao) for 20 min (1 mL/well). Finally, images were acquired with a digital camera to calculate the number of colonies formed in each group. The survival rate of cells in the HCT-8/Vector group was taken as 100% to calculate the survival rate of the cells in the HCT-8/ANRIL overexpression group.

### Transwell assay

Transwell chamber (Corning, Corning, NY, USA) was used to assess cell migration and invasion. In brief, cancer cells and HLECs cultured in tumor culture supernatant (1×10^5^ cells in 200 μL of serum-free RPMI-1640 medium) were seeded in the upper chamber of Transwell plates, and 700 μL of 10% FBS RPMI-1640 medium was added to the lower chamber. After culturing for 18 h, the upper and lower chambers were fixed with 0.2 mL and 0.7 mL of 4% paraformaldehyde for 20 min, respectively, and stained with 0.1% crystal violet for 20 min. The cells in the upper chamber of the Transwell plate were carefully wiped off with a cotton swab. Finally, the cells that migrated were photographed under a phase-contrast microscope (Leica Microsystems GmbH) at ×200 magnification. The number of cells was counted in three random fields.

### 3-(4,5-Dimethylthiazol-2-yl (MTT) assay

HCT-8/ANRIL or HCT-8/Vector cells (1.2×10^5^ cells/mL) were seeded onto a 96-well plate (100 μL/well), and incubated with or without 0.25 mg/mL PZH for 24 h (six replicates in each group). Then MTT solution was added (0.5 mg/mL, 100 μL/well). After incubating at 37 °C for 4 h, dimethyl sulfoxide (100 μL/well) was added, and the absorbance at 570 nm was measured with a microplate reader (Tecan Austria GmbH, Austria).

### Tube formation assay

HLECs cells cultured in the tumor culture supernatant were seeded in a six-well plate (2 mL/well) at a density of 1.0×10^5^ cells/mL and cultured for 24 h. Before the experiment, Matrigel was spread on a 48-well plate (on ice) and then placed in a 37 °C, 5% CO_2_ incubator for 1 h. HLECs were treated during gelation, inoculated on the gel at 2×10^4^ cells/well, incubated for 3 h, and then observed and photographed under a phase-contrast inverted microscope (×100). The percentage of cells forming tubes was calculated in three random fields.

### Statistical analysis

The data in this study were expressed as the mean ± standard deviation, and SPSS version 26.0 was used for statistical analysis. If the data conformed to the normal distribution, Student’s *t* test was used to analyze two groups of data; one-way analysis of variance was used to analyze multiple groups of data, and *P* < 0.05 or *P* < 0.01 was considered statistically significant.

## Supplementary information

Supplementary figure and legend

## Data Availability

The datasets used and/or analyzed during the current study will be available from the corresponding author upon reasonable request.
