# Supplementary material for: ANRIL promotes the regulation of colorectal cancer on lymphatic endothelial cells via VEGF-C and is the key target for Pien Tze Huang to inhibit cancer metastasis
Source: Cancer Gene Ther. 2023 Jun 7;30(9):1260–73. doi: 10.1038/s41417-023-00635-w (PMC10501904; doi:10.1038/s41417-023-00635-w)
Supplement: Supplementary file 1 — Supplementary figure and legend [file 41417_2023_635_MOESM1_ESM.docx]

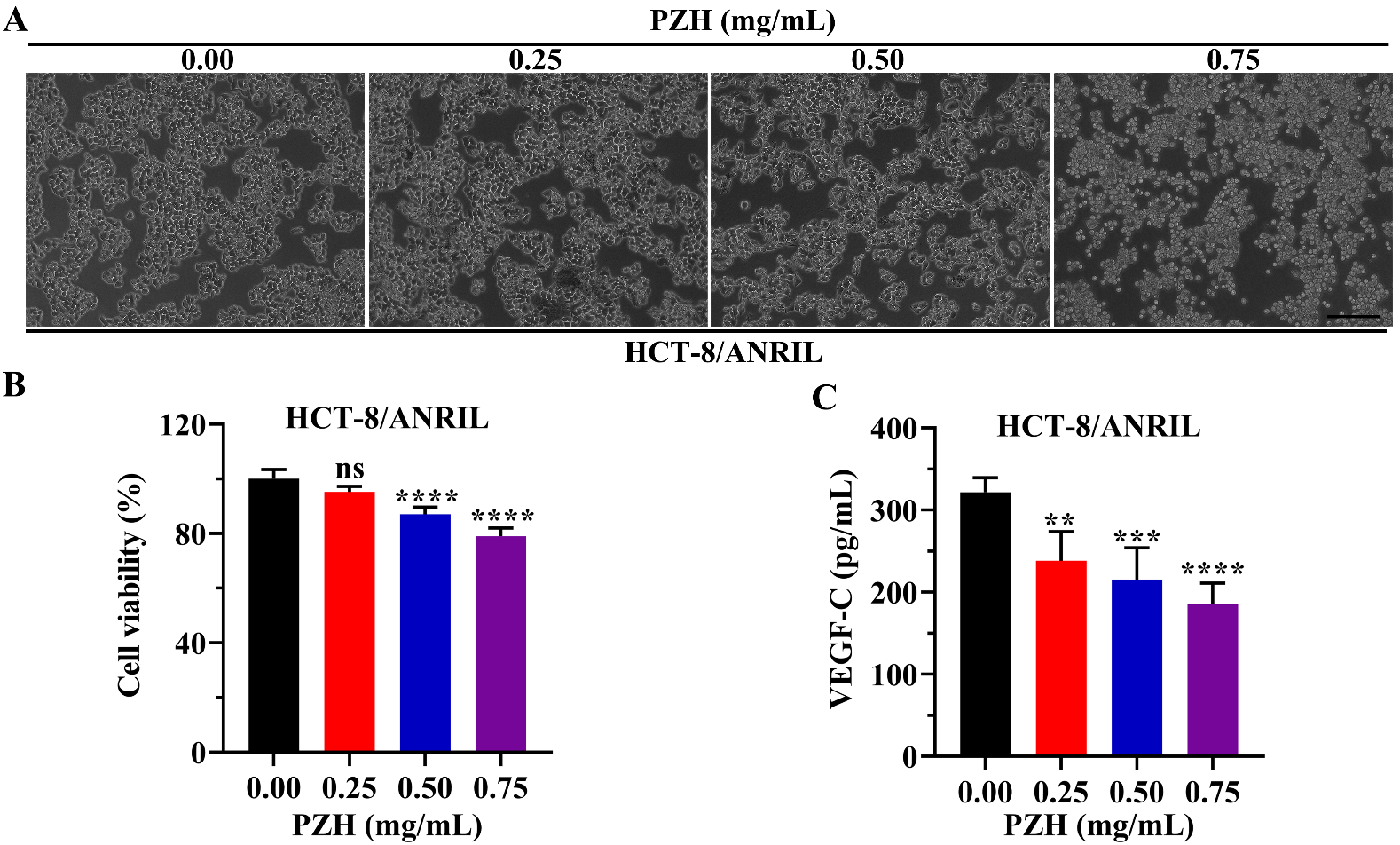


**Supplementary Figure 1.** Effects of different concentrations of PZH on the viability and secretion of VEGF-C of HCT-8/ANRIL cells. **A.** The morphological changes of HCT-8/ANRIL cells with different concentrations of PZH treatment (0.25, 0.5, 0.75 mg/ml). **B, C.** The cell viability and the concentration of VEGF-C in supernatant of HCT-8/ANRIL with different concentrations of PZH treatment, compared with 0.00 mg/mL PZH treatment. Data represent the mean ± SEM of experiments conducted in triplicate. **, *** and ****, p < 0.01.
